# Activity-Induced Enhancement of Superdiffusive Transport in Bacterial Turbulence

**DOI:** 10.3390/mi13050746

**Published:** 2022-05-08

**Authors:** Chenliang Xie, Yanan Liu, Hao Luo, Guangyin Jing

**Affiliations:** School of Physics, Northwest University, Xi’an 710127, China; chenliang.xie@foxmail.com (C.X.); luo@nwu.edu.cn (H.L.)

**Keywords:** microfluidic channel, bacteria, collective motion, superdiffusion

## Abstract

Superdiffusion processes significantly promote the transport of tiny passive particles within biological fluids. Activity, one of the essential measures for living matter, however, is less examined in terms of how and to what extent it can improve the diffusivity of the moving particles. Here, bacterial suspensions are confined within the microfluidic channel at the state of bacterial turbulence, and are tuned to different activity levels by oxygen consumption in control. Systematic measurements are conducted to determine the superdiffusion exponent, which characterizes the diffusivity strength of tracer particles, depending on the continuously injecting energy converted to motile activity from swimming individuals. Higher activity is quantified to drastically enhance the superdiffusion process of passive tracers in the short-time regime. Moreover, the number density of the swimming bacteria is controlled to contribute to the *field* activity, and then to strengthen the super-diffusivity of tracers, distinguished by regimes with and without collective motion of interacting bacteria. Finally, the non-slip surfaces of the microfluidic channel lower the superdiffusion of immersed tracers due to the resistance, with the small diffusivity differing from the counterpart in the bulk. The findings here suggest ways of controlled diffusion and transport of substances within the living system with different levels of nutrition and resources and boundary walls, leading to efficient mixing, drug delivery and intracellular communications.

## 1. Introduction

Diffusion of substances from molecular to macroscopic scale in fluid is a fundamental process and of great importance for chemical reactions, and biomedical and industrial devices. This transport is also crucial for the survival of microorganisms that are thus able to dynamically regulate the living environment throughout all the scales. In order to break through the diffusion limit of random-work process, a variety of organism systems exploit anomalous diffusion, i.e., ballistic or superdiffusion to promote living activity such as quorum sensing [1,2], intracellular chromosome transport [3], cell migration [4], foraging and feeding [5], colony expansion [6], etc. In non-living chaotic flows, the swirls and jets cause the sticking and jumping processes of the immersed tracer particles, then make tracers diffuse much faster by the Lévy flight motion [7,8]. These vortex structures are usually identified to maintain the superdiffusion, which otherwise is brought back to the normal diffusion at a specific time scale [8], tremendously longer than ultrashort hydrodynamic memory time [9,10,11,12,13].

Luckily, for the tracer particles dispersed in biological fluids with living constitutes, there is another time scale that is significantly longer than the time of the momentum damping for diffusive Brownian particles. The swimming bacteria, for example, generate vigorous flows in the surrounding fluids to considerably improve intracellular communications and also expedite the transport of particulate matter around. By increasing the population of swimming units in the experiments and simulation, the long-time diffusion coefficient of tracer particles was confirmed to significantly grow with the concentration of the active swimmers [14,15,16,17,18]. Particularly, the coherent flows generated by the group motion of active swimmers above a critical concentration resulted in the diffusivity rising over concentration either linearly or non-linearly, relying on the type of swimmers and boundary conditions of the confined geometry [15,16,19,20,21,22,23,24]. In the seminal work, Wu and Libchaber found the enhanced diffusion of microspheres emerging in the bacterial bath, with an apparent diffusion coefficient two orders of magnitude larger than that in the pure thermal bath [15]. Extensive investigations have been conducted to illustrate the diffusion enhancement in the living bath, and a transition from ballistic motion to super diffusion at a short time scale, then back to the normal diffusion in a long time regime [17,18,25,26,27,28,29]. Additionally, spacial confinement constrains the motion of bacteria with walls, in which, for example, microfluidic channels can preliminarily change the hydrodynamical boundary condition for the swimming bacteria and the interaction among intra-cells [30,31,32,33,34,35].

In living systems, nutrition and resources are always subject to variation and fluctuation in a dynamic way. Unlike animal social behavior with important relevance of the member activity to the corporative motion in the group [36], microorganisms rely on the diffusion without sensation but suffer more erratic motion from environmental fluctuation. The motility and mobility of the living units therefore either intensify or depress their motions in response to the environmental change. Intuitively, the activity is directly introduced with the free speed of individual bacteria in a dilute suspension of microswimmers. However, when microswimmers become crowded, orientational updates become more frequent due to the interaction among individuals, and then the apparent activity of the individuals is depressed. The activity of the moving units is thus one of the most crucial measures to adjust the diffusion and transport behavior of all the inclusion substances. In this context, the number density of the swimmers with evolving activity is expected to change the background flow and transport of the carrier particles, and also distinct mechanisms can be recognized with or without collective motion due to the strong interaction of the active swimming particles. Indeed, changing the system energy with temperature, interacting rotor particles exhibited ballistic motion at mean filed energy, then superdiffusion process due to Levy-flight de-trapping within overall energy at a moderate level [14]. Interestingly, the activity of the protein motors within the cell plays the fundamental bio-functionality to make life alive, which regulates the chemical reaction at the molecular level and mechanical movement of cellular constitutes, even the cell itself as a whole. It has been reported that by tuning the activity of the microtubule filaments, distinct active turbulence patterns with topological defects were observed, and therefore the ballistic diffusion of tracer particles was recognized within these active nematic systems [37,38]. Although the long-time diffusion dynamics have been extensively investigated for the travel and transport of the passive and active particles in dilute and dense living flows generated by self-driven microorganisms, how the short-time transient behavior with the superdiffusion process quantitatively depends on the control parameters is less examined by well-defined experiments. In particular, an understanding of how the activity of the motile microorganisms, one of the crucial measure of the driving ability of the swimmers plays a role in the superdiffusion strength is still lacking.

Herein, the activity of the bacterial swimmers at the individual level is proposed to regulate the superdiffusive process of the seeded tracer particles. A parameter of activity coefficient is defined to quantify and explore the detailed superdiffusion of seeded tracers before relaxation to the normal diffusion, to say, the short-time diffusion regime. For these purposes, we mainly control oxygen depletion and obtain the various activities on average from individual bacteria in the dilute regime, and the resultant *filed* activity from the collective motion by population bacteria in the dense regime as well. The activity is determined to monotonically enhance the superdiffusion exponent, by the increasingly large fraction of strait motion in the trajectories of the tracer particles. The onset collective motion separates the higher exponent of the superdiffusion from that in the dilute suspension of bacteria. Successful escaping or trapping due to the drag force close to the wall surfaces is additionally checked, from the activity point of view, to distinguish the diffusivity from the one in the bulk correspondingly. Therefore, activity control, one of the fundamental factors for active matter, finds its essential role is to change the living behavior of the organism, and directly contribute to the diffusivity of immersed substance, thus offering the possible method for improving the efficiency of transport and applications in the living system.

## 2. Materials and Methods

### 2.1. Bacteria Culture

The *Bacillus subtilis* (strain 168) are used here as the bacterial swimmers, with the body length and diameter measured as l=4.2±0.4μm and b=0.8±0.1 μm, respectively. The typical swimming speed of free bacteria is about 20 μm/s. Following the standard culturing protocol, stored bacteria at $-20{\;}^{{}^{\circ}}$C is transferred into the Luria–Bertani broth (tryptone 1.0%, yeast extract 0.5%, and NaCl 1.0%, in the unit of *w*/*v*), loaded on the shaker for overnight growth (at the temperature of $30{\;}^{{}^{\circ}}$C, with shaking speed of 200 rpm). Thereafter, the saturation culture is diluted 1:100 in fresh LB culture medium and followed by the growth at $30{\;}^{{}^{\circ}}$C for 6 h until the mid-log phase of growth with the optical density (OD) ∼0.8 (wavelength λ=600 nm). The bacteria are then harvested from the culture medium by centrifugation (1500 g for 5 min). After removing the upper supernatant, the bacteria pellet is resuspended to the minimum medium of motility buffer (MB medium, 0.01 M potassium phosphate, 0.067 M NaCl, and 10 M EDTA, pH 7.0), in which bacteria keep motile but do not divide. Finally, the bacterial suspension was washed once in MB and adjusted to the target volume fraction. Note that the volume fraction of bacteria at the standard concentration $n_{0}\approx 10^{9}$ cells/mL is 0.001 in volume fraction, and OD∼1.

### 2.2. Microfluidic Channel and Imaging Method

**Figure 1:**
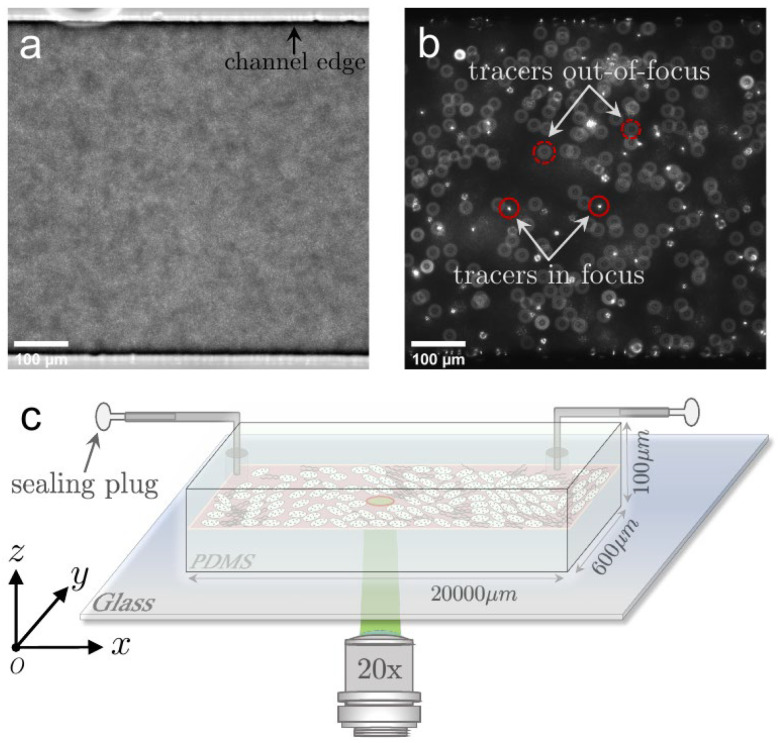
Experimental setup. (**a**) Bright-field snapshot of bacterial suspension in high concentration and (**b**) typical fluorescent snapshot of the tracer particles seeded in bacterial suspension. (**c**) Sketch of PDMS microfluidic channel with the dimension of 600 μm, 100 μm and 20 mm in width, height and total length, respectively. The entrance and outlet of the channel are sealed at the tubing ends after imposing bacterial suspension into the channel.

The prepared bacterial suspension is well mixed with the tracer particles of polystyrene microsphere (1μm in diameter, red fluorescence-labeled, Fluoro-Max, Thermal Scientific, Amarillo, TX, USA), and adjusted to the final concentration of 0.0005% (*w*/*v*). The mixture is then gently imposed into the PDMS channel using a syringe pump, through a metal needle connection (22 ga Luer tubes, PHYMEP, Paris, France), as show in the sketch (Figure 1). Afterwards, both the entrance and exit ends of the channel are blocked for quiescent fluid within the channel. The channel is manufactured with the dimension in the width of w=600μm and height of h=100μm (Figure 1). Since bacteria need to consume oxygen for swimming, the swimming speed (activity) of bacteria in the closed channel will naturally decay over time. Image sequences are recorded under the inverted fluorescence microscope (Nikon Ti2-E, Japan) equipped with the high-speed camera (Hamamatsu, ORCA-Flash4.0 v3), the focal plane is fixed at the mid-height of the channel (z=50μm). Before recording the motion of tracer particles under fluorescence mode, the bright-field images of bacterial suspension in the same view field are simultaneously taken within 10s duration by a 20 × lens (NA = 0.45) with the frame rate of 50 Hz at the resolution of 2048×2044 in pixels. The imaging process starts 3 min after the transfer of the suspension mixture into the PDMS channel, and 500 frames are taken for each image stack with a waiting time period of 10 min. In this way, a variety of different bacterial activities are regulated in our experiments.

### 2.3. Image Processing and Data Analysis

Particle image velocimetry (PIV) and particle tracking velocimetry (PTV) are combined to analyze the image sequences. The turbulent flow field is characterized by the PIV method via Matlab routines (plugin of PIVlabs in Matlab) based on the bright-field image sequence for dense bacteria, which provides the living background flow, advecting and diffusing the tracer particles. For each pair of neighboring frames, the interrogation window and step size are set as 43.52μm and 10.88μm respectively in all PIV processes. The total kinetic energy $E_{xy}\left(x,y,t\right)$ and enstrophy $\Omega_{z}\left(x,y,t\right)$ quantify the energy injection (2D projection) by bacteria. Through the velocity field, $E_{xy}\left(t\right)=\langle \left(v_{x}^{2}\left(x,y,t\right)+v_{y}^{2}\left(x,y,t\right)\right)/2\rangle$ and $\Omega_{z}\left(t\right)=\langle \nabla \times v\left(x,y,t\right)\rangle$, where 〈⋯〉 represents the spatial average, while the time-averaged energy ${\bar{E}}_{xy}$ and time-average enstrophy ${\bar{\Omega }}_{z}$ set the length scale of vortex in bacterial turbulence as $\Lambda ={\left({\bar{E}}_{xy}/{\bar{\Omega }}_{z}\right)}^{1/2}$. On the other hand, the velocity correlation functions offer the spatial and time correlation for the flow structures of the turbulence of bacterial suspension. Calculations are performed as $C_{vv}\left(\delta r\right)={\langle v\left(r\right)\cdot v\left(r+\delta r\right)/v^{2}\left(r\right)\rangle }_{r}$ and $C_{vv}\left(\delta t\right)={\langle v\left(t\right)\cdot v\left(t+\delta t\right)/v^{2}\left(t\right)\rangle }_{t}$, where ${\langle \;\rangle }_{r}$ and ${\langle \;\rangle }_{t}$ represent the spatial and time average. The correlation length λ and time τ can be calculated by fitting the data to $e^{-\delta r/\lambda }$ and $e^{-\delta t/\tau }$.

Meanwhile, the diffusion behavior of microspheres in bacteria suspension is characterized by the PTV analysis. The trajectories of the tracers are extracted from the image sequences by using the TrackMate plugin (Fiji, open-source software, NIH). The object diameter and quality threshold used in TrackMate are 15 pixels (5μm) and 5 respectively. The maximum linking distance of neighboring frames with the LAP Tracker is set to 15 pixels (5μm). The displacements Δx of the microspheres from the trajectories are calculated at different bacterial activities and concentrations, and in the bulk and vicinity of the wall surfaces. Then, the mean square displacement (MSD) of microspheres is derived by $\mathrm{MSD}\left(\Delta t\right)=\langle |r\left(t_{R}+\Delta t\right)-r\left(t_{R}\right){|}^{2}\rangle$, where 〈⋯〉 denotes the ensemble average over all microspheres and reference times $t_{R}$.

## 3. Results

### 3.1. Activity-Induced Enhancement of Superdiffusion

Bacterial turbulence emerges in a bacterial suspension when the bacteria become dense. This chaotic flow contains correlated spatiotemporal motion with vortices, and the strength of spontaneous flow is determined by the motility of swimming bacteria. Within the closed PDMS channel, the oxygen in total is fixed from the beginning and is approximately cut from supply due to the negligible diffusive gas from the ambient compared to the fast consumption by the population of the bacteria. The oxygen control is essentially balanced between the oxygen diffusion flux through the porous PDMS wall into the channel from the ambient and the consumption rate of bacteria. On the one hand, the oxygen flux J=−D∇c due to diffusion is used to estimate the supplying oxygen for bacteria, which is calculated as $1.8\times 10^{19}$ molecules/(m^2^·s), where $\nabla c\approx (c_{a}-c_{s})/H$ is the gradient of oxygen concentration between air $c_{a}=2.7\times 10^{25}$ molecules/m^3^ and suspensions $c_{s}=7.3\times 10^{23}\;\mathrm{molecules}/m^{3}$ over a thickness of H=5 mm of PDMS, and the diffusion coefficient *D* is about $3.4\times 10^{-9}$ m^2^/s. The valid surface area of PDMS is ($S=1.2\times 10^{-5}$ m^2^), the porosity of PDMS is (*p*∼10%) and oxygen solubility is (α∼1%). Thus, the final injection rate by diffusion is $\chi_{1}=J\cdot S\cdot p\cdot \alpha \approx 2.1\times 10^{11}\;\mathrm{molecules}/s$. On the other hand, oxygen consumption rate by bacteria $\chi_{2}=N\kappa \approx 1.2\times 10^{14}\;\mathrm{molecules}/s$ is determined by using total number ($N=nV=1.2\times 10^{8}\;\mathrm{cells}$) of bacteria and consumption rate of oxygen $\kappa =10^{6}\;\mathrm{molecules}/\left(\mathrm{cell}\cdot s\right)$. Obviously, the oxygen consumption rate $\chi_{2}$ by the dense bacteria is much larger than the supply rate $\chi_{1}$ of diffusive oxygen through PDMS walls. Consequently, it is reasonable to neglect the oxygen supply by diffusion through the PDMS channel in our experiments. Therefore, over time, the oxygen concentration supplied for bacteria is monotonically decreasing.

**Figure 2:**
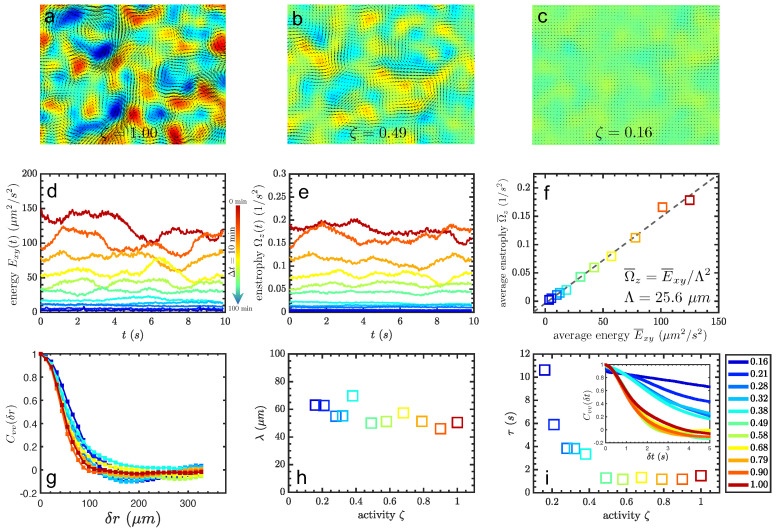
(**a**–**c**): Velocity filed of the bacterial turbulence via PIV analysis. (**d**,**e**): Total kinetic energy and enstrophy of the flow field with decaying activities at each waiting period of 10 min within a total time of 100 min, indicated by the color plots (i.e., red for highest activity, and blue for lowest activity). (**f**): Linear relation between kinetic energy and enstrophy with a slope of $\Lambda^{2}$, corresponding to the square of the typical radius of the vortices. (**g**): Correlation length from velocity field, and its dependence on activity in (**h**). (**i**): Correlation time (inset) vs. activity.

The typical bacterial turbulence is observed at the middle height of the microfluidic channel, and its flow field is characterized through PIV measurement, see the Method Section 2 for details. The activity of the bacteria decreases due to oxygen consumption over time, indicated by the velocity field with arrows and vorticity with colors in Figure 2a–c. A clear decay of motion ability is shown with the shorter arrow, which is defined here as the activity coefficient ζ, quantifying the spatiotemporal kinetic energy on average. This coefficient is derived from spatiotemporal average speed ${\bar{v}}_{i}$ normalized by the one ${\bar{v}}_{0}$ at the initial state of bacterial suspension when transferred into the microfluidic channel, to say, $\zeta ={\bar{v}}_{i}/{\bar{v}}_{0}$. At every waiting period of 10 min within the total time of 100 min in our experiments, the global activity of bacterial turbulence is observed to gradually decay as shown in Figure 2d. Besides, the chaotic behaviors of bacterial collective motions contain typical whirls and jets, which can be reflected by enstrophy strength and are shown in Figure 2e. It turns out that there is a linear relationship between the kinetic energy $E_{xy}$ and enstrophy $\Omega_{z}$, as shown in Figure 2f. The slope of the relation $\Lambda^{2}$ is estimated as the typical radius of vortices in bacterial turbulence around Λ∼25 μm. The typical radius of vortices of bacterial turbulence does not significantly vary with the activity of bacteria, in agreement with previous work [39]. For further confirmation, the correlation length (twice the typical radius of vortices ∼2Λ) of the velocity field is calculated, shown in Figure 2g. The correlation length roughly linearly decays with increasing the activity, from almost 70μm to around 50μm as shown in Figure 2h. This tendency has been overlooked but can be seen carefully from the plot of the correlation function as varying activity in the work [39]. Differently, this tendency is in contrast with the result in the work [40], where the correlation length increased from 10 μm to a plateau value around 40μm in a two-dimensional film with a height of 400μm when the average speed was lower than 20μm/s, which is similar to the case in the current work. The temporal correlation τ also decreases with activity, as shown in Figure 2i. This time correlation is important when arguing the hydrodynamical memory time towards the longer persistent time of the tracer superdiffusion, compared to the extremely short time scale of microspheres in the normal fluid. Combining the tendency of both correlation length and time, it shows that bacteria form larger correlated motions of vortices with smaller motility, which gives a larger correlation length and longer correlation time.

**Figure 3:**
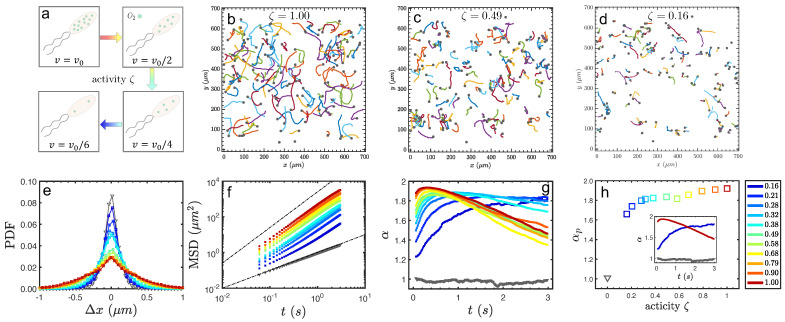
(**a**): Schematic activity decaying is reflected by bacterial velocity. (**b**–**d**): Typical trajectories of tracer particles in bacterial turbulence at different activities, with a black dot as the starting point for each trajectory. (**e**,**f**): Probability density function of the displacement in x-direction Δx at Δt=1/50 s, and MSD = $<\Delta x^{2}\left(t\right)>$ of tracer versus lag time *t*, by varying activity (in color). (**g**): Apparent superdiffusion exponent $\alpha_{p}$ calculated from the slope of time dependence, and (**h**) as a function of activity ζ level.

Due to active energy injected continuously from each living unit, the superdiffusion process of tracers is present for a short time. Here, the fluorescent microspheres are used to characterize the superdiffusion behavior in bacterial turbulence due to the activity effect in a three-dimensional microfluidic channel. The trajectories of tracers in different activities are displayed in Figure 3b–d. It is clearly seen that the average length of trajectories decreases as activity faded away with oxygen consumption. Meanwhile, the trajectories are straighter in a more active case, while more random in a less-active case. The probability distribution functions of displacement along the *x* direction are plotted in Figure 3e at different activity levels (plots in color), which evolve from a Gaussian distribution to a non-Gaussian (exponential distribution, the tail at large |Δx|) in a high activity state. It is obvious that particles in the thermal bath do Brownian motion with $<\Delta x^{2}\left(t\right)>\propto t$. Differently, the particles in bacterial turbulence behave superdiffusively in a short time, around seconds, as MSD$∼t^{\alpha },\;\alpha >1$, and returned to normal diffusion afterwards, as shown in Figure 3f. The time evolutions of the slope α(t) of MSD are plotted with different activities in Figure 3g. At the early stage of the motion, the slope α increases to a peak value and then gradually decays to a plateau with the value of 1 for the normal diffusion. The dark blue curve, denoting the very low activity, slowly approaches the peak value, which seems to saturate at a plateau for too long to be observed due to the issue of being out-of-focus. The peak value of the slope, instead, is used to set the time window, within which the apparent superdiffusion exponent $\alpha_{p}$ is calculated from the curve fitting of MSD. As shown in Figure 3h, the apparent superdiffusion exponent $\alpha_{p}$ monotonically increases with the activity strength.

In brief, the activity of individual bacteria by controlling the oxygen consumption determines the intensity and characteristic scale of the global motion of group bacteria. As activity increases, there is a clear decay of correlation length and time in the vigorous bacterial turbulence. For particles seeded in the chaotic flow, they perform a non-Gaussian distribution of displacement and present a significantly superdiffusive signature in a short time scale. Therefore, the activity level of the living constituted within the suspension promotes the transport efficiency for seeded tracers, with an approximately monotonical enhancement of the superdiffusion exponent.

### 3.2. Superdiffusion in Dilute and Crowed Bacterial Suspensions

**Figure 4:**
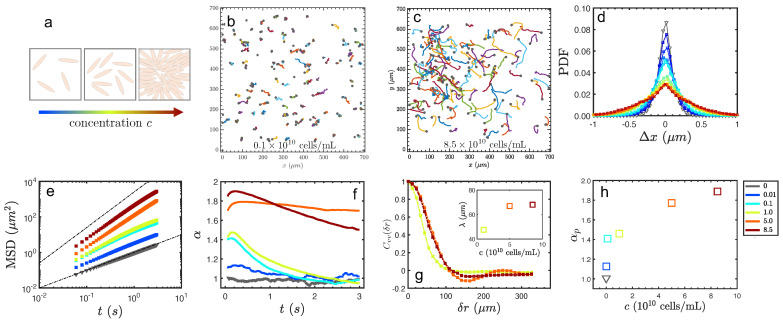
(**a**): Sketch of increasing bacterial population. (**b**,**c**): Typical trajectories of tracer particles in bacterial suspension at different number densities. (**d**–**f**): PDF of Δx at Δt=1/50 s, MSD = $<\Delta x^{2}\left(t\right)>$ of tracer versus lag time *t*, and Slope of MSD in bacteria suspension with varying number density (in color). (**g**): Correlation and characteristic length (inset) of the velocity field at high number density of bacteria. (**h**): Apparent superdiffusion exponent $\alpha_{p}$ vs. number density, calculated from the slope of time dependence.

Individual bacteria, as an example of self-propelled particles, continuously transfer chemical energy into kinetic motion and stir background fluid to flow. Multiple bacteria align themselves due to hydrodynamic interaction and steric effect and start the correlated motions with chaotic-like vortices above a critical number density. In the context of the activity above, the concentration of bacteria therefore definitely changes the strength of input energy, sketched in Figure 4a. Hence, the activity variations by varying bacteria concentrations, change the extent of the tracer superdiffusion. The typical trajectories of particles in bacteria by varying the number density of bacteria are shown in Figure 4b,c. For the dilute case $n=0.1\times 10^{10}$ cell/mL, the trajectories are short and random, whereas, at high density, the trajectories are straighter due to the apparent advection by the collective motion of the bacteria. The displacements along with the *x*-direction exhibit Gaussian distributions for cases with a low confrontation of bacteria, while the long tail of non-Gaussian distribution occurring at the highest concentration, shown in Figure 4d. For cases lower than $n=1\times 10^{10}$ cells/mL, the slope of the MSD returned to normal diffusion within 3 s. It is clear that active energy injected into the system at even a very low level can push the particles to perform super-diffusion in a short time (Figure 4e). However, the superdiffusion exponents drastically jump to a large value and decay back to normal diffusion in a considerably longer time, as shown in Figure 4f, at high bacteria concentrations in which collective motion is present (Figure 4g). Following the same rule discussed above, the apparent superdiffusion exponent $\alpha_{p}$ also increases with number density *c*, as shown in Figure 4h. For the cases with collective motion, as number density increases, the correlation length also increases from 40μm to around 70μm.

In short, the concentration of bacterial suspension distinguishes the diffusion of tracer particles into two types, i.e, shorter superdiffusion in dilute suspension and persistent superdiffusion in bacterial turbulence (dense bacteria). The presence of the collective motion makes the superdiffusion stronger, with a long relaxation to normal diffusion, compared to the sudden dropping of superdiffusion in dilute cases. The concentration effect is reflected by the activity level, measuring the injected energy from the bacteria, and the superdiffusion strength is correlated to this activity adjustment of the swimmer number confined within the channel.

### 3.3. Wall Effect on Superdiffusion

**Figure 5:**
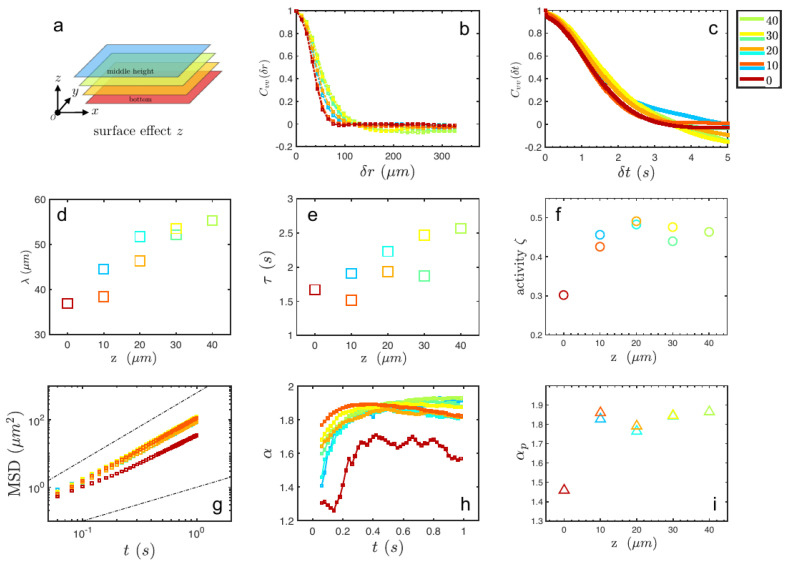
(**a**): Representation of layer scanning along the vertical direction in the PDMS channel. (**b**,**c**): Spatial and temporal correlation of the velocity field at a different height in every 10 μm. (**d**–**f**): Correlation length, time, and activity depend on the height within the channel, respectively. (**g**–**i**): MSD = $<\Delta x^{2}\left(t\right)>$ of tracer, Slope of MSD versus lag time *t*, and apparent superdiffusion exponent $\alpha_{p}$ by varying height, respectively.

The bacteria used in this work continuously rotate their slender bodies and multiple bundled helical flagella in opposite directions, exerting forces on the background fluid explained by a hydrodynamic force-dipole model. It is also well accepted that swimming bacteria have hydrodynamic interaction with each other as well as solid boundaries thus leading to distinct individual and collective behaviors. Hence, the boundary effect on group motion, and therein particle diffusion is considered by varying the observation plane from the middle height of the channel to the lower wall surface sketched in Figure 5a. As shown in Figure 5b,c, the correlation functions both in space and time are analyzed, indicating a linear decreasing correlation length from 55μm to 35μm as well as a correlation time in the order of seconds when approaching the wall. Note that, for this series of experiments, the starting activity of bacteria is set to 50% of the initial activity of fresh bacteria, i.e., ζ=0.5 is used as the starting activity level. The later variation of the activity comes from the contribution of the boundary effect. Note also that as the observation layer is varied, the number density of the bacteria suspension is fixed to be $8\times 10^{10}$ cells/mL, producing the collective motion. During the continuous scanning across the height, the activity decays from ζ= 0.5 to 0.2 as the distance decreases from half-height to the bottom wall. The solid bottom wall with a non-slip boundary suppresses the motion of bacteria as well as the tracer particles. From the MSD curves in Figure 5g, tracer particles resting in different layers experience superdiffusion during the whole observation period. The time evolutions of the slope of MSD α are shown in Figure 5h, exhibiting a tendencey to rise and later fall, far away from reaching the normal diffusion α=1. The apparent exponent $\alpha_{p}$ shows a stable value of 1.8 in the bulk as shown in Figure 5i. On the layer very close to the wall, the boundary effect significantly constrains the bacterial motion and the particle diffusion as well with a clear decay of diffusion ability $\alpha_{p}$∼1.45. In other words, the solid boundary suppresses the activity of individual and collective bacteria thus weakening the superdiffusion of tracer particles.

## 4. Discussion

### 4.1. Oxygen-Depletion Induced Activity Variation

Intuitively, it seems easy to control the swimming speed of the bacteria or the activity of the swimmers, and thus regulate the agitation intensity of the seeded tracer particles from the disturbing background flows. In reality, it is a bit tricky to utilize the controllable release of nutrients or drugs in the culture medium, and even more difficult to synchronize the activity of the whole colony or population of bacteria. As an aerobic species, *Bacillus subtilis* increasingly consume oxygen when it becomes more crowded in the suspension. Within a PDMS channel, the bacteria are sheltered to the state of collective motion and slowed down due to the consumption of the limited oxygen until the complete depletion. Diffusion $D_{c}$ of the oxygen molecules, consumption of oxygen by bacteria with number density of *n* at rate of κ, and the convection term v·∇c by heterogeneous velocity v of flow, all contribute to the change rate $c_{t}=\partial c/\partial t$ of the oxygen concentration c(t) as [41]:

(1) $$c_{t}+v\cdot \nabla c=D_{c}\nabla^{2}c-n\kappa f\left(c\right),$$

where *f*(*c*) is constant. The simulation showed that both bacteria and oxygen concentration rapidly reached the steady-state and presented the uniform distribution across the channel height with a thickness of less than 200μm [42]. So, the vigorous mixing by collective motion homogenizes the oxygen across the total height of the fluidic channel (h=100μm) in the present work. Therefore, the chemotactic migration of bacteria in the vertical direction is negligible. Different from the controlling of the external oxygen supplying into the bacterial suspension [40], here the oxygen consumption by the dense bacteria naturally degrades the motility and then the activity of individuals. The activity continuously decreased with time after the transfer of dense bacteria into the channel. Ten minutes for each time period is set to reach a specific activity in sequence. The result shows a monotonically decaying of activity over time. Eventually, the lowest mean velocity of the coarse-grained bacterial suspension is stopped at $8\%\;v_{0}$ in our experiments.

### 4.2. Superdiffusion as the Cross-Over Regime

In the thermal bath, the tiny molecules independently collide with Brownian particles with extremely high frequency and lose memory very fast, which is a so-called collision of Gaussian white noise. In order to visualize the ballistic and superdiffusion of the Brownian particles with a mass of $m_{p}$ seeded in the normal liquid, an ultrafast imaging method is required to capture the trajectory before the transition to the normal diffusion at a characteristic time $\tau_{p}=m_{p}/\left(6\pi \eta a\right)$ about $10^{-6}$ s [10,11,12,13]. For particles in a bacterial suspension, there is another time scale significantly longer than the time of the momentum damping for classic Brownian particles, i.e., the correlation time of a spontaneous flow generated by swimming bacteria, which promotes the correlated motion of the carried tracer particles with the aid of the living hydrodynamic memory. This hydrodynamic memory time can be reflected by the correlation time of the flow field of bacterial chaotic flows in Figure 2i, showing the time scale of 1∼10 s. This time scale is in agreement with the cross-over time from superdiffusion to normal diffusion. Experimentally, the longer hydrodynamic memory indeed maintain superdiffusion regime with longer duration before the transition to the normal diffusion in our measurements (Ref. to Figure 3f and Figure 4e), which is consistent with measurements for the system of *E. Coli* bacterial suspension [15]. Inspired by the theoretical analysis by Wu and Libchaber [15], an additional white noise term is introduced into the Langevin equation, then:

(2) $$m_{p}\ddot{r}=-\gamma \dot{r}+f_{w}\left(t\right)+f_{c}\left(t\right),$$

where the Stokes friction coefficient reads as γ=6πηa, and the symbol of over dot represents the time derivative on the position r of the tracer. Since the superdiffusion has an exponent 1<α<2, the superdiffusion process is conceptually decomposed by ballistic motion (α=2) and normal diffusion (α=1). This decomposition allows the correspondence to the different types of noises, so to speak, the color noise $f_{c}$ responsible for ballistic motion and the white noise $f_{w}$ responsible for normal diffusion. Therefore, statistically, the relations of the noise are written as $<f_{w}\left(t\right)>\;=\;0$, $<f_{w}\left(t\right)\cdot f_{w}\left(0\right)>\;=\;\sqrt{k_{B}T\gamma }\delta \left(t\right)$, and $<f_{c}\left(t\right)>\;=\;0$, $<f_{c}\left(t\right)\cdot f_{c}\left(0\right)>\;=\;4D_{0}\gamma^{2}\exp\left(-t/\tau \right)/\tau$. By this crude approximation, only the color noise term $f_{c}$ is considered; the vortices have a characteristic length comparable to the root mean square displacement, i.e., $\Lambda ∼{\left(<{\left[\delta r\right]}^{2}>\right)}^{1/2}∼4D_{0}t{\left[1-\exp\left(-t/\tau \right)\right]}^{1/2}$, which was about 10μm for bacterial suspension of *E. Coli*. With the same order of magnitude in our case (Figure 2f), the correlation length of the vortices in the bacterial turbulence of *Bacillus Subtilis* is about 50μm.

We suspect that the fractional part *f* of ballistic motion with straight displacement raises the superdiffusion exponent α→2, whereas the pure normal diffusion (1−f) lets α→1. The weighted sum of the Gaussian and Laplace (exponential) distribution can be roughly used to fit the probability density function of the displacements, then the simplest empirical relation for the apparent exponent of superdiffusion is about α=2·f+1·(1−f)=1+f. Qualitatively, the non-Gaussian process due to high activity raises the proportion of the straight motion of tracer particles, therefore increasing the exponent $\alpha_{p}$ shown in Figure 3h. The time correlation of tracer particles is written as $\tau ∼D_{t}/<v^{2}>$, which presents the time duration for tracer particles to perform persistent directed motion before reaching the long-time diffusive motion at diffusivity constant of $D_{t}$ [24]. Then the correlation length is $l_{t}∼l^{1-\beta }L^{\beta }$, which means the correlation length in our case weakly depended on the activity of swimming bacteria (Figure 2f,h) since both the size of the system and the unit cell are fixed. As shown in Figure 3g, it is interesting that superdiffusion is eventually brought to normal diffusion after a sufficiently long time; however, motion in a bacterial suspension with low activity needs a much longer time to be relaxed to pure diffusion.

### 4.3. Activity Effect

The activity changes due to swimmer concentration and drag friction from the wall surface, which thereafter is relevant to the flow instability and then the tracer diffusivity. Each swimming bacteria with non-zero speed contributes to active stress and can be approximately written as:

(3) $$\sigma ∼f_{a}l\left(p_{i}p_{j}-\frac{1}{3}\delta_{ij}\right)\propto \zeta l\left(p_{i}p_{j}-\frac{1}{3}\delta_{ij}\right),$$

where the simplification of the linear relation of active force $f_{p}$ on the swimming speed $\bar{v}=\zeta v_{0}$ is considered, and activity $\zeta =\bar{v}/v_{0}$ here is defined as the normalized mean speed to the free swimming speed $v_{0}$ of a single bacterium. The magnitude of the active stress is correlated to the activity defined in the present work, as $\left|\sigma \right|∼\eta \zeta v_{0}l^{2}$ by the straightforward dimensional analysis. A similar introduction of the activity coefficient was used as the definition of the active stress towards the instability of an active system [21].

In the active nematic system, Sanchez et al. tuned the activities of microtubule filaments by controlling the concentration of ATP as fuel converted to mechanical energy into the filament suspension via the molecular motor proteins [37]. At a different level of activity, a transition occurred from the ballistic motion of tracer particles seeded in the filament suspension to the superdiffusion, corresponding to cases with high and low activity, respectively. The detailed motion and diffusion processes in our bacterial suspension are different from those observed in the microtubule suspension with nematic order and dynamic formation (and annihilation) of topological defects. However, the activity-enhanced diffusion in our bacteria suspension is in agreement with the result in the microtubule system. Moreover, the velocity correlation at the characteristic length is also consistent with prediction in the theory and simulation conducted in ref. [38]. The injected energy related to the activity of the bacterial swimmers is eventually dissipated into the surroundings, and the balance of injection and the dissipation sets a velocity scaling as $v∼\zeta \lambda (p_{i}p_{j}-\frac{1}{3}\delta_{ij})$. When the number density of the swimmers is constant due to suppression of growth and dividing process of the swimmers, the collective motion thus shows weak dependence on the oxygen concentrations, and slightly decreases with the activity similar to the observation in ref. [39]. The correlation length is larger for the low activity of the swimmers; then the superdiffusion is kept longer before recovering the normal diffusion.

Recently, by Lagrangian statistics of the tracking particle in living turbulence, the activity parameter was introduced in the phenomenological continuum theory, where a stable superdiffusion regime was realized with the diffusion exponent of 4/3 lasting for several time decades at very high activity [43]. With the activity defined here, the phenomenological continuum equation can be written as:

(4) $$\partial_{t}u+\lambda u\cdot \nabla u=-\nabla p-\Gamma_{0}\nabla u-\Gamma_{2}\nabla^{4}{u+(\zeta -\beta |u|}^{2})u,$$

Numerically, varying the activity coefficient ζ in the work by Mukherjee et al., the long persistent time of the superdiffusion process was evident when the activity became larger, which is very similar to our experimental results. However, in their report, the ballistic transport at a short time rapidly transforms to the normal diffusion with a remarkably narrow time window for the superdiffusion [43]. In comparison, here, lowering the activity of the bacteria by oxygen depletion, the superdiffusion is maintained for a much longer time than we can access in our observation.

### 4.4. Concentration Effect

Individual *Bacillus Subtilis* generate a flow field captured by a force-dipole model based on the hydrodynamic singularity method. On the other hand, population bacteria, *pushers* in particular, are able to generate living turbulence with vortices at a length scale larger than their body size. As a consequence, the interaction between the swimming organisms themselves inevitably plays a role in the tracer motion. Enhanced diffusivity of the tracer particles was proposed as the empirical dependence on the concentration $n_{b}$ and speed $\bar{v}$ swimmers as $D_{n}-D_{0}=\beta \bar{v}n_{b}$, compared with the thermal diffusivity of the tracers $D_{0}$ [25,44,45,46]. In other words, the flux of the active swimmer was determined to linearly contribute to the diffusivity of the immersed passive particles.

Interestingly, the increase of the swimmer density strongly randomized the orientational ordering and therefore lowered the diffusivity of swimmers themselves with linear relation in the simulation, while significantly increasing the diffusivity of tracers [15,16,22,24]. With the calculation of the time correlation of the swimming bacteria within the collective motion, it turns out that the persistent time of correlation is 1∼10 s in the order of magnitude (Figure 2i), which is a little bit larger than that of the *running* time of isolated bacteria. The ballistic motion of the transported tracer particle occurs and keeps the straight path during a short time regime, which however is not the case in the dilute bacterial suspension. The superdiffusion exponent is far less than 2 and drops to the value of 1 (i.e., normal diffusion), implying the hydrodynamic interaction from multiple bacteria interrupts the ballistic motion of tracer particles.

### 4.5. Wall Effect

Boundary walls with viscous friction are expected to regulate the field of the spontaneous flow generated by the bacteria and therefore change the activity of the individual swimmers. Intuitively, confined space constrains the flow in a different way compared with that in 3D space. The non-Gaussian distribution of the tracer displacements was determined to be dependent on swimmer number density and was quantified by a fractional number between the Gaussian and Laplace distributions of the tracer displacements [17]. However, the non-Gaussian dynamics of the tracer motion still possessed the normal diffusion even at a short-time regime in a bath filled with the ‘puller’ swimmers, while superdiffusion was found for tracer motion kicked by the ‘puller’ swimmers with the significantly enhanced diffusion process when confined to a 2D case [18]. Strong drag resistance at the wall surface in our case, to some extent, suppresses the collective motion and superdiffusion, which, here, is ascribed to the activity reduction. However the wall effect is complex in that the collective motion of the microorganisms does drive the motion of the tracers to the superdiffusive process despite the strong viscous resistance of the non-slip hypothesis, and even puller swimmers incapable of forming the correlated flows also promote the superdiffusion of the tracers by long-range hydrodynamical interaction with wall surfaces when confined into, for example, a 2D space [18].

## 5. Conclusions

In summary, a PDMS microfluidic channel with a regular rectangle cross-section is used to quantify the tracer motion in bacterial suspension with the dynamics of abnormal diffusion. The flow field of bacterial suspension and transport trajectory are characterized in fine detail for the seeded particles, when varying the activity of bacteria under control of oxygen consumption over time. It is found that there is a non-negligible growth of the correlation between the length and time of the collective motion and activity, which decreases with time. The tracer particles behave in superdiffusion and the extent of the superdiffusion rises sharply in low activity and slightly grows with a plateau in the high activity regime. The tunable concentration of bacterial suspension is devoted to tracer diffusion in the active bath with or without apparent collective motion. Tracer particles are subject to superdiffusion in bacterial suspension even with very dilute active swimmers. Whereas significant enhancement of superdiffusion occurs for tracers in the dense bacterial bath with continuous collective motions, this superdiffusion maintains for much longer than the memory time of hydrodynamic coherence of the bacterial turbulence. In the end, the boundary effect is examined by comparing the superdiffusion strength at a different layer in the channel, showing that the solid wall weakens the motion of the bacteria and reduces the activity of collective motion thus the ability of particle superdiffusion. The tuning oxygen flux would be more powerful by designing the branching PDMS channels, and a sophisticated oxygen gradient to precisely control the activity of the bacteria in further investigations. In reality and applications, it would be very interesting to control the supply of oxygen, nutrition, or antibiotic drug for the purpose of bacterial motility control. The optimal conditions for the activity value of the microorganisms would depend on the detailed applications and industrial usages with the specific mixing time. In general, the flux of gas flow, the concentration of nutrition gradient and concentration of antibiotics are good control parameters from the biochemical point of view. On the other hand, the permeability and thickness of the PDMS channel are also interesting control parameters, from the materials’ point of view. Additionally, the wettability and roughness of the channels and tubes are practical designs considering the hydrodynamic boundary controls.

## Data Availability

Not applicable.

## Sample Availability

The authors declare no sample availability.
